# Statistical Methods for Mapping Multiple QTL

**DOI:** 10.1155/2008/286561

**Published:** 2008-06-08

**Authors:** Wei Zou, Zhao-Bang Zeng

**Affiliations:** ^1^Bioinformatics Research Center, North Carolina State University, Raleigh, NC 27695, USA; ^2^Department of Statistics, North Carolina State University, Raleigh, NC 27695, USA; ^3^Department of Genetics, North Carolina State University, Raleigh, NC 27695, USA

## Abstract

Since Lander and Botstein proposed the interval mapping method for QTL mapping data analysis in 1989, tremendous progress has been made in the last many years to advance new and powerful statistical methods for QTL analysis. Recent research progress has been focused on statistical methods and issues for mapping multiple QTL together. In this article, we review this progress. We focus the discussion on the statistical methods for mapping multiple QTL by maximum likelihood and Bayesian methods and also on determining appropriate thresholds for the analysis.

## 1. INTRODUCTION

Quantitative genetics studies the variation of quantitative traits and their genetic basis. When R. A. Fisher laid down the basic theoretical foundations of quantitative genetics, the focus of study was to partition the overall variation into genetic and environmental ones. With the development of polymorphic markers for many species, current research interest is to partition genetic variation to individual quantitative trait loci (QTL) in the genome as well as interaction among them [1]. A QTL is a chromosomal region that is likely to contain causal genetic factors for the phenotypic variation under study.

The basic principle of QTL mapping has been established in Sax'swork [2] work in beans. If there is a linkage disequilibrium (LD) between the causal factor and a marker locus, mean values of the trait under study will differ among subject groups with different genotypes at the marker locus [3]. Though this idea is still directly used in certain settings (e.g., LD-based QTL mapping in unrelated human), the advance of QTL mapping methodology has allowed simultaneous use of multiple marker information to improve the accuracy and power to estimate QTL locations and effects. Lander and Botstein [4] presented a likelihood-based framework for interval mapping (IM), where the putative QTL genotype was conditional upon a pair of flanking markers' genotypes as well as the phenotype. A least square equivalence of IM [5] was also proposed where phenotypic values were regressed onto expected genetic coefficients of a putative QTL. Motivated by the conditional independency between marker genotypes, composite interval mapping [6] proposed to introduce additional flanking markers as covariates into the likelihood function to reduce the confounding effects from nearby QTL when scanning the current interval. However, most of these methods were still designed to detect a single QTL at a time based on a statistical test that a candidate position for a QTL has significant effect or not. The test was constructed to test each position in a genome and thus created a genome scan for QTL analysis.

Though intuitive and widely used, these methods are still insufficient to study the genetic architecture of complex quantitative traits that are affected by multiple QTL. When a trait is affected by multiple loci, it is more efficient statistically to search for those QTL together. Also in order to study epistasis of QTL, multiple QTL need to be analyzed together. In this setting, QTL analysis is basically a model-selection problem. In this paper, we discuss recent research progress and outstanding statistical issues associated with mapping multiple QTL in experimental cross populations.

## 2. MULTIPLE INTERVAL MAPPING (MIM)

Multiple interval mapping is targeted to analyze multiple QTL with epistasis together through a model selection procedure to search for the best genetic model for the quantitative trait [1, 7, 8].

For *m* putative causal genes for the trait, the model of MIM is specified as

(1) $$y_{i}=u+\overset{m}{\underset{r=1}{\sum }}\alpha_{r}x_{ir}^{*}+\overset{t}{\underset{r\neq s\subset (1,\ldots ,m)}{\sum }}\beta_{rs}(x_{ir}^{*}x_{is}^{*})+e_{i},$$

where

- *y*_*i*_ is the phenotypic value of individual *i*, *i* = 1, 2,…, *n*;
- *u* is the mean of the model;
- *α*_*r*_ is the main effect of the *r*th putative causal gene, *r* = 1,…, *m*;
- *x*_ir_* is an indicator variable denoting genotype of the *r*th putative causal gene, which follows a multinomial distribution conditional upon flanking marker genotypes and genetic distances;
- *β*_*rs*_ is the possible epistatic effect between the *r*th and the *s*th putative causal genes, assuming there are *t* such effects;
- *e*_*i*_ is an environmental effects assumed to be normally distributed.

As shown by Kao and Zeng [7], Kao et al. [8], given a genetic model (number, location, and interaction of multiple QTL), this linear model suggests a likelihood function similar to that in IM but with more complexity. An expectation/maximmization (EM) algorithm can be used to maximize the likelihood and obtain maximum likelihood estimates (MLE) of parameters.

The following model-selection method is used to transverse the genetic model space in QTL cartographer [1, 9, 10].

1. Forward selection of QTL main effects sequentially. In each cycle of selection, pick the best position of an additional QTL, and then perform a likelihood ratio test for its main effect. If a test statistic exceeds the critical value, this effect is retained in the model. Stop when no more QTL can be found.
2. Search for epistatic effects between QTL main effects included in the model, and perform likelihood ratio tests on them. If a test statistic exceeds the critical value, the epistatic effect is retained in the model. Repeat the process until no more significant epistatic effects can be found.
3. Reevaluate the significance of each QTL main effect in the model. If the test statistic for a QTL falls below the significant threshold conditional on other retained effects, this QTL is removed from the model. However, if a QTL is involved in a significant epistatic effect with other QTL, it is not subject to this backward elimination process. This process is performed stepwisely until no effects can be dropped.
4. Optimize estimates of QTL positions based on the currently selected model. Instead of performing a multidimensional search around the regions of current estimates of QTL positions, estimates of QTL positions are updated in turn for each region. For the *r*th QTL in the model, the region between its two neighbor QTLs is scanned to find the position that maximizes the likelihood (conditional on the current estimates of positions of other QTL and QTL epistasis). This refinement process is repeated sequentially for each QTL position until there is no change on estimates of QTL positions.

An important issue in model selection is the significance level to include or eliminate effects. In regression analysis, such threshold is usually decided based on information criteria, which has the following general form

(2) $$IC=-2\left(\log L_{k}-\frac{kc(n)}{2}\right),$$

where *L*_*k*_ is the likelihood of data given a genetic model with *k* parameters, *c*(*n*) is a penalty function and can take a variety of forms, such as,

- *c*(*n*) = *log*⁡(*n*), which is the classical Bayesian information criterion (BIC);
- *c*(*n*) = 2, which is Akaike information criteron (AIC);
- *c*(*n*) = 2*log*⁡(*log*⁡(*n*));
- *c*(*n*) = 2*log*⁡(*n*);
- *c*(*n*)=3*log*⁡(*n*).

When the penalty for an additional parameter in the model is low, more QTL and epistatic effects are likely to be included in the model. Thus it is particularly important to determine an appropriate penalty function for model selection.

Sequential genome scans require detectable main effects for the components of interaction effect. An alternative approach, exhaustive search of all marker combinations, is a computational and statistical problem even in two dimensions. From a yeast eQTL mapping data with over 6000 expression traits and 112 individuals [11], Storey et al. [12] showed that the sequential search was more powerful than exhaustive one to detect pair-wise QTL main effects and interaction effects. However, in a different setting using simulations under a series of quantitative trait model assumptions, Evans et al. [13] showed that the exhaustive search can be more powerful than the sequential one with over 1000 individuals in the mapping population and a Bonferroni correction for 100 000 tests. The inconsistency is partially related to sample size. A larger sample can make unadjusted *P* values more significant. Witte et al. [14] showed that the required sample size increases linearly as the number of tests increases logarithmically with a simple Bonferroni correction.

## 3. THRESHOLD TO CLAIM QTL

We need to decide the threshold for declaring QTL from the profile of test statistics across the genome. General asymptotic results for regression and likelihood ratio tests are not directly applicable for genome scans given the large number of correlated tests performed in the scans and the limited sample size.

### 3.1. Type I error rate control

When markers are dense and the sample size is large, Lander and Botstein [4] showed that an appropriate threshold for LOD score was (2log10)*t*_*α*_, where *t*_*α*_ solves the equation *t*_*α*_ = (*C* + 2*G* *t*_*α*_)*χ* ^2^(*t*_*α*_). *C* is the number of chromosomes of the organism. *G* is the length of the genetic map, measured in Morgans. *χ* ^2^(*t*_*α*_) is the probability that a random variable from a *χ*_1_^2^ distribution is less than *t*_*α*_.

Churchill and Doerge [15] proposed a method based on permutation tests to find an empirical threshold specifically for a QTL mapping study. Data were shuffled by randomly pairing one individual'sgenotypes with another'sphenotypes, in order to simulate the null hypothesis of no intrinsic relationship between genotypes and phenotypes. Thus, this method takes into account sample size, genome size of the organism under study, genetic marker density, segregation ratio distortions, and missing data.

According to Churchill and Doerge [15], the genome-wide threshold to control type I error rate for mapping a single trait can be found in the following procedure.

1. Shuffle the data *N* times by randomly pairing trait values with genotypes. When there are multiple traits under study, these phenotypes should be shuffled together to keep their correlation structure.
2. Perform mapping analysis and obtain the maximum test statistic in each of *N* shuffled data. This provides an empirical distribution *F*_*M*_ of the test statistic for the genome scan at the null.
3. The 100(1 − *α*) percentile of *F*_*M*_ will provide an estimated critical value.

This permutation procedure is equivalent to the Bonferroni correction for multiple testing when test statistics are independent. Suppose there are *n* such statistics *t*_*i*_ (for *i* = 1,…, *n*) from a null distribution *F*. *F*_*M*_(*T*), the distribution function of maximum of the *n* statistics, can be expressed as Pr(max(*t*_*i*_) < *T*) = *F*(*T*)^*n*^. When we find a threshold *T*, such that Pr(max(*t*_*i*_) > *T*) = 1 − Pr(max(*t*_*i*_) < *T*) ≤ *α*, it is equivalent to require 1 − *F*(*T*)^*n*^ = 1 − (1 − Pr(*t*_*i*_ > *T*))^*n*^ ≤ *α*, or Pr(*t*_*i*_ > *T*) ≤ *α*/*n*, the Bonferroni adjusted threshold. When test statistics are correlated, the permutation method provides a threshold that is less than that from Bonferroni.

A related permutation procedure was also suggested by Doerge and Churchill [16] for mapping procedures that QTLs are declared sequentially using a forward selection procedure. Two methods were suggested to find a genome-wide threshold for the second QTL while controlling effects of the first QTL.

- Conditional empirical threshold (CET). Mapping subjects are put into blocks according to the genotype of the marker identified as (or closest to) the first QTL. Permutation is applied within each block. Following the procedure described above by Churchill and Doerge [15], maximal test statistic of each genome scan is collected and CET is obtained. One problem of CET is that markers linked to the first QTL will continue to show assocation with the trait variation as in the original data. To avoid CET being elevated by such markers, it is suggested to exclude the complete chromosome where the first QTL is located when collecting null statistics.
- Residual empirical threshold (RET). The residues from the genetic model with the first QTL are used as new phenotypic values to be permuted. Maximal null statistics from genome-wide scans are then collected to find RET.

Applied to multiple interval mapping, Zeng et al. [1] also suggested to use a residual permutation or bootstrap test to estimate appropriate threshold for the model selection in each step of sequential test. In this test, after fitting a model with identified QTL effects, residuals of individuals are calculated and permutated or bootstrapped to generate a null model for the conditional test to identify additional QTL. This threshold is more appropriate for the conditional search, but computationally more intensive.

### 3.2. Score-statistic-based resampling methods

Permutation is a computationally intensive method for generating empirical threshold. Zou et al. [17] suggested that the genome-wide threshold could be more efficiently computed based on score statistic and a resamplng method. A score statistic can be computed at each genome position. If we multiply a score function by a standard normal random variable with mean zero and variance one, the resulting score statistic mimics that under the null hypothesis. Thus by multiplying a number of standard normal variables, we can very efficiently generate an empirical distribution of score statistic under the null. This method is flexible and can be used to test a null hypothesis in a complex model. A similar algorithm was also suggested by Seaman and Müller-Myhsok [18]. Conneely and Boehnke [19] extended the approach by replacing the resampling step with a high dimensional numerical integration.

Unlike these approaches, Nyholt [20] suggested another method that addresses the multiple testing issue: the number of independent tests across the genome can be approximately estimated as a function of eigenvalues derived from the correlation matrix of marker genotypes.

### 3.3. False discovery rate (FDR)

In QTL mapping, as marker coverage in a genome increases, it is less likely that a casual variant is not in LD with any marker and may be missed. On the other hand, the number of markers showing significant correlation with the phenotype by chance is also expected to grow, if the type I error rate for each test is controlled at a preset level. To handle this multiple testing problem, stringent family-wise control of type I error is usually applied, which is designed to control the probability of making at least one false discovery in a genome-wide test. However, a more powerful approach may be to control false discovery rate (FDR) [21], or to control the expected proportion of false discoveries among all the markers passing a threshold. This essentially allows multiple false positive declarations when many “significant” test statistics are found. Such a relaxation is driven by the nature of the problem under study. “It is now often up to the statistician to find as many interesting features in a data set as possible rather than test a very specific hypothesis on one item” [22].

According to the notation from Storey [22], Table 1 shows the possible outcomes when *m* hypotheses *H*_1_, *H*_2_,…, *H*_*m*_ are tested. For independent tests, Benjamini and Hochberg [21] provided a procedure (known as linear step-up procedure or BH procedure) to control expected FDR, that is, *E*[(*V*/*R*) ∣ *R* > 0] × Pr(*R* > 0) at the desired level *α*(*m*_0_/*m*) or *α* (since *m*_0_ is generally unknown, a conservative up-bound estimate *m*_0_ = *m* was used) as follows:

- sort *P* values from the smallest to the largest such that *P*_(1)_ ≤ *P*_(2)_,…, *P*_(*m*)_;
- starting from *P*_(*m*)_, compare *P*_(*i*)_ with *α*(*i*/*m*);
- let *k* be the first time *P*_(*i*)_ ≤ *α*(*i*/*m*), reject all *P*_(1)_ through *P*_(*k*)_.

Benjamini and Yekutieli [23] showed that “if test statistics are positively regression dependent on each hypothesis from the subset corresponding to true null hypotheses (PRDS), the BH procedure controls FDR at level *α*(*m*_0_/*m*).” For QTL mapping, PRDS can be interpreted as follows [24]: if two markers have correlated allele frequencies and neither is related biologically to the trait, test statistics associated with the two markers should be positively correlated. Such positive correlation is intuitively correct and supported by simulation results [24].

To check the performance of BH procedure on FDR control in genome-wide QTL scan for a single trait, Sabatti et al. [24] considered a simulated case-control study in human. Three susceptibility genes were simulated to affect the disease status. The genes were assumed to be additive and located on different chromosomes. The results showed that the BH procedure can control the expected value of the FDR for single-trait genome-wide scan. For multiple-trait QTL analysis, Benjamini and Yekutieli [25] considered 8 positively or negatively correlated traits. Using simulation, they showed that BH approach seemed to work for multiple trait analysis too.

According to Benjamini and Yekutieli [25], to control FDR for QTL analysis in each trait at level *α* does not always mean that the overall FDR for these multiple traits is also *α* : if there are *k* independent and nonheritable traits, the overall FDR should be 1 − (1 − *α*)^*k*^ ≈ *k* *α*. It is safer to control FDR for all the tests simultaneously.

Yekutieli and Benjamini [26] suggested to make use of dependency structure in data, rather than treat them as annoying cases. They expected an increase of testing power when using an empirical true null statistic distribution instead of assuming some theoretical ones to get *P* values. Empirical null distributions are used extensively in pFDR and local FDR as discussed below.

Though BH approach is a handy and intuitive tool, it should be used with caution when applied to QTL mapping. First, BH approach controls the expected value of FDR. Simulation studies showed that the actual FDR for a particular QTL mapping dataset can be higher [24]. Second, FDR = *E*[(*V*/*R*) ∣ *R* > 0] Pr(*R* > 0) may be tricky to interpret when Pr(*R* > 0) is far below than 1. Weller et al. [27] are the first ones to apply FDR criteria in QTL mapping area. They claimed that 75% of the 10 QTL declared in their study were probably true by controlling FDR at 25% using BH approach. Zaykin et al. [28] however objected to the interpretation because *E*[(*V*/*R*) ∣ *R* > 0] could be much higher than FDR = 25% when Pr(*R* > 0) was much smaller than 1. It is *E*[(*V*/*R*) ∣ *R* > 0], also known as pFDR discussed in Section 3.4, that really contains the information about the proportion of false discovery. Weller [29] further argued that if one assumes *R* follows a Poisson distribution, Pr(*R* > 0) should be very close to 1 when they observed *R* = 10. In later FDR literature, the assumption that Pr(*R* > 0) ≈ 1 has been widely adopted [25, 30].

### 3.4. Positive discovery rate (pFDR)

pFDR, or *E*[(*V*/*R*) ∣ *R* > 0], was considered less favorable than FDR [21] without the additional term Pr(*R* > 0): we cannot decide an arbitrary threshold *α*, 0 < *α* < 1, and guarantee that pFDR ≤ *α* regardless of the actual proportion of true null hypothesis in all tests. For example, when *m*_0_/*m* = 1, pFDR is always 1 and cannot be controlled at a small *α*. In this case, however, FDR = pFDR × Pr(*R* > 0) can be controlled at *α* by reducing the rejection region to push Pr(*R* > 0), and then FDR, towards *α*. Thus, Benjamini and Yekutieli [25] considered that pFDR should be only estimated after a fixed rejection threshold was decided; or in QTL mapping, pFDR should estimate (instead of control) the proportion of true/false QTL, after the null hypothesis was rejected at *R* linkage peaks by a certain rule (e.g., a type-I error control procedure).

From the discussion in Section 3.3 we know that allowing Pr(*R* > 0) much smaller than 1 might bring trouble in interpreting the results; and when *m*_0_/*m* ≈ 1, we might want to see an FDR measure that is close to 1 [31]. This helped to bring up the interest in pFDR. Storey [31] presented a Bayesian interpretation of pFDR based on *P* values. Assuming there are *m* tests *H*_1_, *H*_2_,…, *H*_*m*_ with associated *P* values *P*_1_, *P*_2_,…, *P*_3_, and

- *H*_*i*_ = 0 denots the *i*th null hypothesis is true, and *H*_*i*_ = 1 otherwise;
- to model the uncertainty in each hypothesis test, each *H*_*i*_ is viewed as an identical and independent random variable from a Bernoulli distribution with Pr(*H*_*i*_ = 0) = *π*_0_ and Pr(*H*_*i*_ = 1) = *π*_1_ = 1 − *π*_0_;
- *P*_*i*_ follows a distribution *F*_0_ if *H*_*i*_ = 0 and *F*_1_ if *H*_*i*_ = 1; it follows a mixed distribution unconditionally: *F*_*m*_ = *π*_0_ *F*_0_ + *π*_1_ *F*_1_;
- Γ = [0, *γ*] is the common rejection region for all *H*_*i*_,

then

(3) $$\begin{array}{cc} \text{pFDR}(\gamma ) & =\mathrm{Pr}(H_{i}=0\;|\;P_{i}\in \Gamma ) \end{array}$$

(4) $$\begin{array}{cc} & =\frac{\mathrm{Pr}(H_{i}\;=\;0)\mathrm{Pr}(P_{i}\;\leq \;\gamma \;|\;H_{i}\;=\;0)}{\mathrm{Pr}(P_{i}\;\leq \;\gamma )\mathrm{Pr}(R\;>\;0)} \end{array}$$

(5) $$\begin{array}{cc} & \approx \frac{\pi_{0}\gamma }{R/m[1-{\left(1-\gamma \right)}^{m}]}. \end{array}$$

Notice that, when *π*_0_ = 1 and *m* is reasonably large, formula (5) is roughly equal to *γ*(*m*/*R*), which is the BH approach to estimate FDR. We can also see from formula (5) that instead of assuming *π*_0_ = 1, a data-driven estimate of *π*_0_ will give a smaller pFDR estimate or a more powerful procedure if *π*_0_ is estimated smaller than 1. Efron et al. [32] gave an upper bound of *π*_0_ by assuming an “accept region” *A* = [*λ*, 1] such that Pr(*H* = 0 ∣ *P* ∈ *A*) ≈ 1:

(6) $$\frac{\int_{A}f_{m}(z)dz}{\int_{A}f_{0}(z)dz}=\frac{\int_{A}[\pi_{0}f_{0}(z)+(1-\pi_{0})f_{1}(z)]dz}{\int_{A}f_{0}(z)dz}\geq \frac{\int_{A}\pi_{0}f_{0}(z)dz}{\int_{A}f_{0}(z)dz}=\pi_{0},$$

where *f*_0_, *f*_1_, *f*_*m*_ are the density functions of *F*_0_, *F*_1_, *F*_*m*_, respectively. The left-hand side of this equation is equivalent with the following estimator [30]:

(7) $$\pi_{0}=\frac{\#\{P>\lambda \}}{m(1-\lambda )}.$$

These formulas lead to *q* value, the minimal pFDR when rejecting a hypothesis with *P* value *P*_*i*_. This approach is more powerful than the BH approach [21] in genomics applications. An R package “*q* value” is available to convert a list of *P* values to *q* values [30]. The *q* value approach can actually find Γ while controlling a specific pFDR rate besides finding pFDR given Γ. However, as discussed above, FDR control in QTL mapping should be applied with caution.

When *P*_*i*_ are identical and independent distributed, pFDR becomes identical to the definition of proportion of false positives (PFP) [22, 33]:

(8) $$\begin{array}{cc} \text{PFP} & =\frac{E(V)}{E(R)} \end{array}$$

(9) $$\begin{array}{cc} & =\frac{\sum_{i=1}^{m}\gamma \,\mathrm{Pr}(H_{i}=0)}{\sum_{i=1}^{m}[\gamma \,\mathrm{Pr}(H_{i}=0)+\mathrm{Pr}(H_{i}=1)\mathrm{Pr}(P_{i}\leq \gamma \;|\;H_{i}=1)]} \end{array}$$

(10) $$\begin{array}{cc} & =\frac{\sum_{i=1}^{m}\pi_{0}}{\sum_{i=1}^{m}[\pi_{0}+\pi_{1}\left(\mathrm{Pr}(P_{i}\leq \gamma \;|\;H_{i}=1)/\mathrm{Pr}(P_{i}\leq \gamma \;|\;H_{i}=0)\right)]}, \end{array}$$

PFP is also estimated similarly as pFDR (cf. formula (5) [33]:

(11) PFP^=π0γR/m.

Contrary to FDR discussed in Section 3.3, PFP has a property that when it is controlled at *α* for each of *n* sets of independent tests, the overall PFP is still *α*. *P*_*i*_ from different sets of tests can have different distributions. In this case, PFP can be different from pFDR [33].

Currently available procedures to control or estimate pFDR or PFP may have variable utility in various mapping designs. Chen and Storey [34] noted that the threshold to control FDR at marker level from one-dimensional genome scan for a single trait could be “dubious” because FDR is affected by the marker densities along the chromosomes. Since a true discovery is to claim a QTL at a marker which is in strong LD with a causal polymorphism, markers that are in strong LD with the true discovery can be additional true discoveries. Thus, FDR decreases when we genotype more markers around a true linkage peak. Using simulations, Chen and Storey [34] showed that the threshold is obtained by controlling FDR varied with the marker density. However, in real applications, people generally consider all markers surrounding a test statistic peak as parts of one QTL, rather than distinct positive discoveries. On the other hand, we can still estimate FDR from *P* values at linkage peaks for different traits that pass certain cutoff value. This is a common situation from an expression QTL study where thousands of traits are analyzed together.

Zou and Zuo [35] showed that family wise error rate control via Bonferroni correction can be more powerful than PFP control. In their simulation, they assumed 1 to 5 true non-null hypotheses out of 1000 independent tests, corresponding to *π*_1_ ≤ 0.5%, which might be too pessimistic for certain QTL mapping studies. As we can see from formula (10), when *π*_1_ is so small, Pr(*P*_*i*_ ≤ *γ* ∣ *H*_*i*_ = 1)/Pr(*P*_*i*_ ≤ *γ* ∣ *H*_*i*_ = 0) has to be very large so that its product with *π*_1_ is considerably larger than *π*_0_ and there is an acceptable PFP level. When the density function of *P*_*i*_ ∣ *H*_*i*_ = 1 is monotonously decreasing in [0, *γ*], which is quite common in reality, *γ* has to be very small to increase the ratio of power over type-I error. Thus, it is not surprising that such a *γ* from PFP would result in a more stringent test than that under Bonferroni correction. [36] made a similar argument and pointed out that family wise error rate control was effective when *π*_1_ was relatively low, and PFP or pFDR approach can be more powerful when *π*_1_ was high. Again, expression QTL studies stand out as an example when pFDR is more favorable: in the yeast eQTL mapping data [11], Storey et al. [12] estimated that *π*_1_ = 0.85 among the genome-wide maximal test statistic for each expression trait.

### 3.5. Local FDR

The Bayesian interpretation of pFDR extends naturally to local FDR [32], denoted as FDR here,

(12) $$\begin{array}{cc} \text{FDR}(T_{i}) & =\mathrm{Pr}(H_{i}=0\;|\;T_{i}) \end{array}$$

(13) $$\begin{array}{cc} & =\frac{\pi_{0}f_{0}}{\pi_{0}f_{0}\;+\;\pi_{1}f_{1}}=\frac{\pi_{0}f_{0}}{f_{m}}, \end{array}$$

where *T*_*i*_ is the test statistic associated with *H*_*i*_; *H*_*i*_ = 0 denotes that the *i*th null hypothesis is true, and *H*_*i*_ = 1 otherwise; *T*_*i*_ has a density *f*_0_ if *H*_*i*_ = 0, and *f*_1_ if *H*_*i*_ = 1; or *f*_*m*_ = *π*_0_ *f*_0_ + *π*_1_ *f*_1_ when hypotheses are mixed together.

There is great similarity between formula (3) and formula (12). Actually Efron [37] showed that *q* value associated with *T*_*i*_ is equivalent with *E*_*t*≥*T*_*i*__FDR(*t*). Storey et al. [12] showed that FDR could be estimated within each cycle of a sequential genome scan for thousands of expression traits; then the average of the FDR in the rejection region was an estimate of pFDR.

The key part in estimating FDR is to estimate *f*_0_/*f*_*m*_. It is possible to assume certain standard distribution under the null hypothesis as *f*_0_ and estimate *f*_*m*_ from nonparametric regression [37]. The following procedure is modified from [12, 32]:

1. permute data under null hypothesis *B* times, and obtain test statistics *z*_*ij*_, *i* = 1,…, *m*, *j* = 1,…, *B*;
2. estimate *f*_0_/*f*_*m*_ from *T*_*i*_ and *z*_*ij*_ (see below);
3. estimate *π*_0_ using formula (7).

*f*_0_/*f*_*m*_ is estimated in the following way:

1. pool all *T*_*i*_ and *z*_*ij*_ into bins;
2. create an indicator variable *y*, let *y* = 1 for each *T*_*i*_ and *y* = 0 for each *z*_*ij*_, thus, Pr(*y* = 1) = *f*_*m*_/(*f*_*m*_ + *B* *f*_0_) in each bin;
3. obtain a smooth estimate of Pr⁡^(y=1) in each bin from an overall regression curve across bins, by combining natural cubic spline with generalized linear models for binomially distributed response variables;
4. equate $\hat{\mathrm{Pr}}(y=1)$ with *f*_*m*_/(*f*_*m*_ + *B* *f*_0_), and get a moment estimate of *f*_0_/*f*_*m*_.

It is noticed that *H*_*i*_ and its associated *T*_*i*_ or *P*_*i*_ are assumed to be from a mixture distribution in both pFDR and FDR estimation. Thus, as pointed out by Storey [22], there is a connection between multiple hypothesis testing and classification. For each test, the test procedure is to classify *H*_*i*_ as 0 or 1, or accepted or rejected. Classification decisions can be made based upon *T*_*i*_, with a rejection region Γ: if *T*_*i*_ ∈ Γ, we classify *H*_*i*_ as 1.

## 4. BAYESIAN METHODS

Bayesian QTL mapping methods try to take full account of the uncertainties in QTL number, location, and effects by studying their joint distributions. Such a method takes the prior knowledge about these parameters as a prior distribution, reduces the uncertainty by integrating the information from the data, and expresses the remaining uncertainty as a posterior distribution of parameters.

Satagopan et al. [38] illustrated the use of Markov chain Monte Carlo (MCMC) algorithm to generate a sequence of samples from the joint posterior distribution of QTL parameters conditional upon the number of QTL. Gibbs sampling algorithm approximates the joint distribution by sampling each parameter in turn, conditional upon the current values of the rest of parameters. Conjugate priors can be chosen so that most of these conditional distributions have explicit distribution functions and can be sampled directly. Otherwise, Metropolis-Hastings algorithm is to be used. Point estimates of individual parameters are obtained by taking the averages of the corresponding marginal distributions. Confidence intervals are obtained as high posterior density regions.

Sen and Churchill [39] proposed to sample QTL genotypes from their posterior distribution conditional upon marker genotypes and trait phenotypes. This multiple imputation method offers a framework to handle several issues in QTL mapping: nonnormal and multivariate phenotypes, covariates, and genotyping errors. It differs from usual MCMC procedures in that a two-step approach is used: first, QTL genotypes are sampled conditioning only on marker genotypes; then, weights can be calculated as the likelihood of the phenotypes given a genotype. These genotypes and weights are then used to estimate posterior distributions.

In both studies, MCMC simulations were conditional upon the number of QTL (*L*) underlying the phenotype. Different values of *L* were tried and Bayes factors were used to decide which value of *L* was more plausible. Bayes factor is the ratio of the probability density of data under two hypotheses. It is a likelihood ratio in some simple cases. In Bayesian QTL mapping, however, likelihoods are weighted by prior distributions of parameters under different hypotheses to get Bayes factors [38].

The development of reversible jump MCMC algorithm [40] suggested one way to treat *L* as a parameter and generate its posterior distribution. Satagopan and Yandell [41] introduced this method to QTL mapping: when updating the current value of *L*, a single QTL may be added or deleted from the model. Yi et al. [42] extended this method to model interacting QTL by allowing 3 types of transdimensional exploration: a main effect or epistatic effect, a single QTL with its effects or a pair of QTL may be added or deleted in one updating step. However, they also observed low acceptance rates for transdimensional jump proposals and hence slow mixing of the markov chains and high computational demand associated with the algorithm.

Yi [43] introduced composite model space approach as a unified Bayesian QTL mapping frame work. The approach incorporates reversible jump MCMC as a special case, and turns the transdimensional search into a fixed-dimensional one. The central idea is to use a fixed length vector to record the current locations of *L* putative QTL and another indicator vector to record whether a QTL'smain or interaction effect is included or excluded. These two vectors decide the actual number of QTL. The fixed length sets an upper bound for the number of detectable QTL. This approach has been implemented in the R/qtlbim package [44, 45].

Without considering computational efficiency, the upper bound can actually be fairly large. Unlike multiple regression analysis based upon least square criteria, which gets in trouble when the number of explanatory variables is larger than the number of observations, Bayesian analysis can handle a large number of explanatory variables through a large number of cycles within each step of Gibbs sampling. Xu [46] showed an example where markers across genome were used simultaneously in Bayesian linear regression and suggested that the Bayesian method could result in model selection naturally: markers with weak effects are “shrunk” so that their posterior mean effects are around zero, markers with strong effects remain strong.

A closely related procedure is ridge regression, where the parameters to an usual linear model *Y* = *X* *β* + *ϵ* are estimated as $\hat{\beta }=(X^{T}X+K{)}^{-1}X^{T}Y$ (*K*=*λ* *I*, *ϵ*∼*N*(0, *σ* ^2^ *I*), *I* is an identity matrix). The estimator turns out to be identical to a Bayesian one which assumes that *β* has a prior normal distribution *N*(0, *σ* ^2^/*λ*) [47]. Ridge regression was initially applied to marker-assisted selection by Whittaker et al. [48] to reduce the standard errors in estimation. Xu [46] found ridge regression was not applicable to genome-wide markers simultaneously because each marker demand a different *λ* in its prior distribution variance. This problem of ridge regression can be fixed in a generalized ridge estimator where *K* is a diagonal matrix with different diagonal elements *λ*_1_, *λ*_2_,… [49]. However, an iterative procedure is required to find these *λ*, which is similar to the MCMC sampling in Bayesian analysis.

A Bayesian QTL mapping analysis usually results in a posterior distribution over the QTL model space. Numerical characteristics from such a distribution provide estimates for parameters. Such an estimation is based upon entire model space, weighted by the posterior probabilities, and hence is more robust than usual MLE. In terms of hypothesis test, Bayes factors, together with prior odds, are used to compare two hypotheses. Unlike *P* values, Bayes factors are calculated under both competing hypotheses; like *P* values, they have to be compared with some commonly used cutoff values (like 0.05 for *P* values) to decide which hypothesis to prefer [50].

There is very clearly a null hypothesis and an alternative hypothesis in single QTL scan [4]. In model selection for a multiple QTL model, however, the number of hypotheses in the model space is huge and the data may well support many models for a complex trait. Posterior distribution emphasizes such uncertainty. Picking a better supported model to interpret mapping results may not fully convey the uncertainty of inference. On the other hand, a pragmatic mathematical model may choose to simplify the complexity and present an inference with basic structural characteristics. See Kass and Raftery [50] for more discussion.

Bayesian QTL mapping does not have the multiple testing issue discussed above. Bayesians agree with the idea to require very strong evidence to call QTL from genome. However, they believe that the reason is that the genome is so large that the prior probability that any one variant or variant combination is causative is very low. Thus, in Bayesian QTL mapping, the multiple testing issue is handled by the low prior density on any one marker or low prior odds for any one hypothesis; and such a stringent requirement is recommended even when exploring a very limited QTL model space unless there is strong prior knowledge against that [51].

## 5. CONCLUSION

To document the evolution of the statistical approaches for QTL mapping, we attempt to follow some threads of methodological development on multiple QTL mapping, threshold determination, and Bayesian QTL mapping methods. We see that this area has been advanced greatly by the interaction between genotyping technologies and statistical methodologies in the last several years, and will continue to be so in the future. The availability of efficient computational algorithms/softwares is essential to the QTL mapping community. However, it is equally important that these tools are applied with thorough understanding of the genetic data and the tools themselves.

## Figures and Tables

**Table 1:** Possible outcomes from *m* hypothesis tests.

	Accepted null	Rejected null	Total
Null true	*U*	*V*	*m*_0_
Alternative true	*T*	*S*	*m*_1_
	*W*	*R*	*m*
